# A comparative analysis of lesional skin, sentinel flap, and mucosal biopsies in assessing acute face transplant rejection

**DOI:** 10.3389/fimmu.2025.1562024

**Published:** 2025-04-01

**Authors:** Martin Kauke-Navarro, Lioba Huelsboemer, Felix J. Klimitz, Fortunay Diatta, Leonard Knoedler, Samuel Knoedler, William J. Crisler, Stav Brown, Christine G. Lian, Federico Repetto, Rachael A. Clark, George F. Murphy, Christine Ko, Bohdan Pomahac

**Affiliations:** ^1^ Department of Surgery, Division of Plastic Surgery, Yale School of Medicine, New Haven, CT, United States; ^2^ Department of Dermatology, Brigham and Women’s Hospital, Harvard Medical School, Boston, MA, United States; ^3^ Program in Dermatopathology, Department of Pathology, Brigham and Women’s Hospital, Harvard Medical School, Boston, MA, United States; ^4^ Department of Dermatology, Yale School of Medicine, New Haven, CT, United States

**Keywords:** face transplantation, vascularized composite allotransplantation (VCA), rejection monitoring, rejection, immunology

## Abstract

**Background:**

Face transplant rejection is primarily monitored through skin biopsies, but mucosal tissue may detect immune rejection events missed by skin biopsies.

**Methods:**

We retrospectively reviewed 47 paired mucosal and facial skin biopsies and 37 paired facial skin and sentinel flap biopsies from nine face transplant recipients. Rejection was graded using the 2007 Banff classification. Correlation, sensitivity, and specificity metrics were assessed.

**Results:**

Mucosa and facial skin rejection grades correlated strongly (r = 0.72, p < 0.0001), with mucosa showing a negative predictive value (NPV) of 0.85 for facial skin rejection. Mucosal biopsies identified rejection in 10 cases missed by facial skin biopsies. Sentinel skin biopsies had high correlation but an NPV of 0.76, missing 24% of rejection cases.

**Conclusion:**

Mucosal biopsies tend to capture the full spectrum of rejection, whereas skin biopsies alone may miss important rejection events occurring in the mucosa. Mucosal biopsies should be integrated into routine monitoring alongside skin biopsies, as they not only sensitively function as sentinel tissue but also provide critical insights into rejection activity that may otherwise go undetected. This dual approach could improve overall transplant surveillance. Inconsistencies in rejection patterns between the two tissues highlight the need for a reworked grading system.

## Introduction

Vascularized Composite Allotransplantation (VCA) involves the transfer of a tissue composite, often including skin, mucosa, adipose tissue, muscle, bone, vasculature, lymphatics and nerves, from a donor to a recipient to replace a complex functional unit such as the hand or face (1, 2). It has emerged as a transformative treatment for patients with severe injuries, with nearly 300 procedures performed worldwide in the past two decades, including over 50 face transplants (fVCA) (3). Despite the clinical success of achieving functional and aesthetic restoration of the most complex defects, acute cellular rejection (ACR) and its sequelae (chronic rejection [CR] with tissue fibrosis and immunosuppression related side effects) continue to limit progress of the field (4–6).

To date, rejection has been incompletely defined and categorized in facial transplants. It is assumed that the immunologic activity in small punch biopsies of facial skin represents the status in all of the tissues that comprise the complex allograft. However, recent evidence from lymphatic and mucosal tissues suggests that skin may not be a reliable proxy for the degree of rejection affecting all tissues constituting face transplants (7–10). In addition to skin and thus unlike limb transplants, the face uniquely contains a second squamous epithelial-lined surface tissue (mucosa) which is readily accessible for serial sampling in addition to skin. Mucosal tissue assessment has not been included in the Banff classification, but the importance of mucosa has recently been highlighted and grading of rejection in mucosal tissue is based on the principles applied to skin grading as published by Bergfeld et al. (11) Similar to skin, rejection is based on the degree of immune cell infiltration and immune mediated injury to keratinocytes of the epithelial layer.

Beyond facial skin and mucosa, sentinel flaps have historically been used to aid in diagnosing rejection. However, data remains limited concerning their correlation with facial skin biopsies and their independent clinical utility. To address this gap, herein we have reviewed our experience with skin, mucosal, and sentinel flap biopsies to better assess rejection patterns and outcomes.

## Methods

### Patient cohort

Nine face transplant patients were included in this study who received 10 fVCAs at Brigham and Women´s Hospital, Harvard School of Medicine. The patients are currently followed at Yale New Haven Hospital (Yale School of Medicine, CT, USA). The study was approved by the local IRBs (2019P002841 at Brigham and Women´s Hospital; 2000030847 at Yale New Haven Hospital). We retrospectively reviewed patient charts and recorded all encounters with paired facial skin and mucosal biopsies and paired facial skin and sentinel flap biopsies. Biopsies taken at the time of known infection such as cellulitis/mucositis or systemic viremia (e.g., CMV) were excluded. Whenever infection was suspected clinically, PAS-D, Gram, AFB, and methenamine silver stain were performed. Samples with positive results were excluded. Clinical data was recorded, including signs of rejection (for example, erythema, ulceration, or change from baseline in presentation of the skin or mucosa) and immunosuppression-related details. Clinical rejection was defined as Banff **≥** II with clinical signs, and subclinical rejection is defined as Banff **≥** II without clinical signs.

### Immunosuppressive regimen

Immunosuppression and infection prevention were carried out following our institution’s protocols (1, 9, 12, 13). In summary, patients received induction therapy with 1 g Mycophenolate mofetil (MMF), 1.5 g Methylprednisolone over 3 days, and 1.5 mg/kg daily of rabbit anti-thymocyte globulin (ATG) for 4 days. Triple maintenance immunosuppression included MMF 2 g/day, Tacrolimus (with target levels of 10–15 ng/mL for postoperative months (POM) 0–6, 8–12 ng/mL for POM 7–12, and 6–10 ng/mL thereafter), and Prednisone. MMF and corticosteroids were withdrawn whenever possible. Sirolimus was administered to two patients who developed side effects from Tacrolimus maintenance therapy. Acute T-cell mediated rejection (TCMR) episodes were typically managed with adjustments in maintenance immunosuppression, steroid pulses, topical therapy (e.g., creams and mouthwash), or ATG/alemtuzumab for refractory TCMR. Anti-humoral therapy for antibody-mediated rejection (AMR) included plasmapheresis, IVIG, eculizumab, and bortezomib, either alone or in combination in one patient. Vancomycin and cefazolin were administered perioperatively for infection prophylaxis, while Micafungin was used for antifungal prophylaxis. Post-transplant, antimicrobial treatment was adjusted according to donor and recipient cultures. Prophylaxis against *Pneumocystis jirovecii* and cytomegalovirus (CMV) infection typically involved 6 months of trimethoprim-sulfamethoxazole (TMP) and valganciclovir, respectively (13).

### Rejection assessment

**Figure 1:**
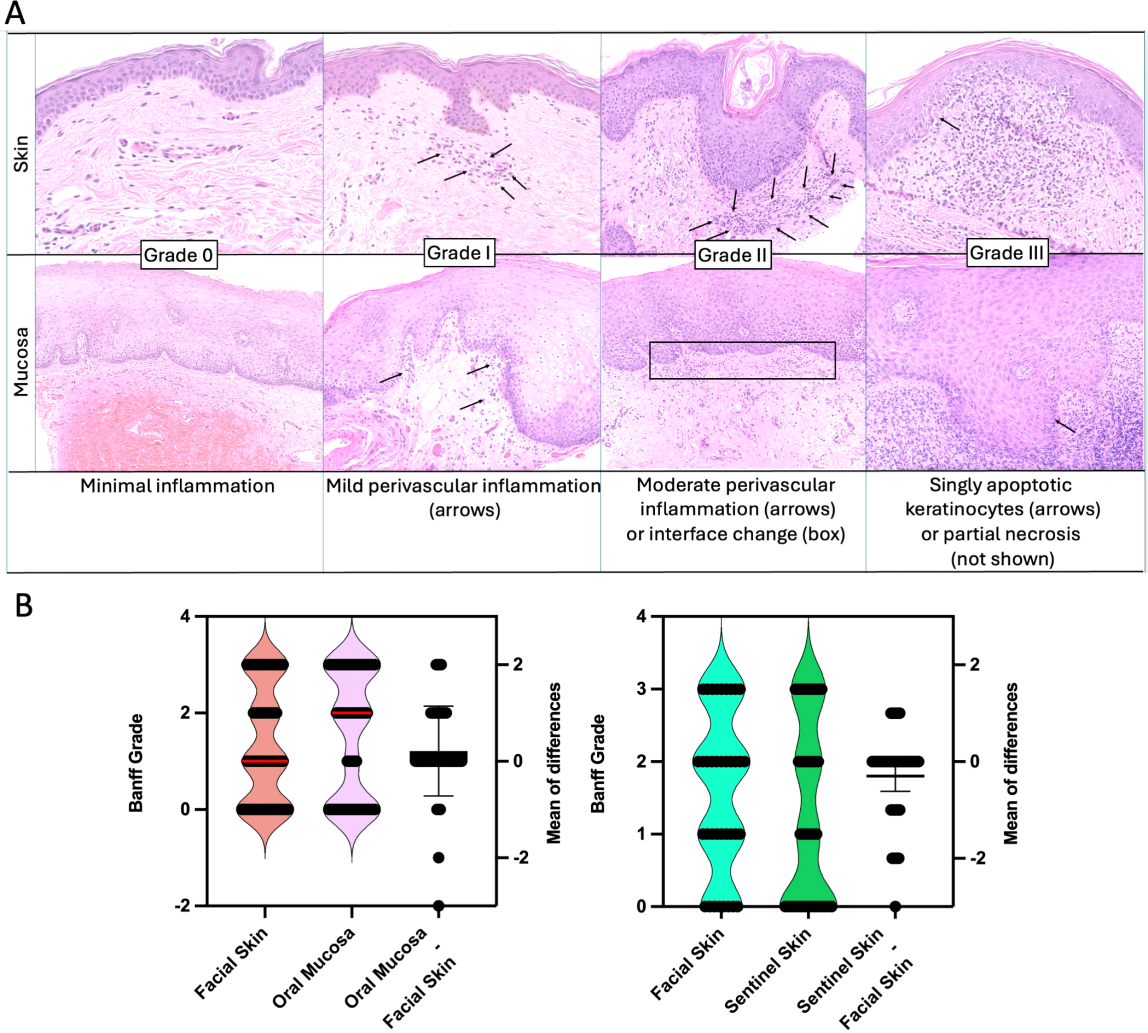
Rejection in skin and mucosa. **(A)** Histological features of acute rejection in skin and mucosa are classified using the Banff grading system (0–III), highlighting similarities between both tissues. Grade 0 indicates minimal inflammation. In both tissues, Grade I is marked by mild perivascular inflammation. Grade II involves moderate perivascular inflammation or interface changes, which may occur with or without spongiosis. Grade III is characterized by the presence of apoptotic keratinocytes in both skin and mucosa. **(B)** Corresponding rejection pairs of facial skin and oral mucosa (left) and facial skin and sentinel skin (right Skin versus Oral Mucosa. The oral mucosa tends to have higher grades of rejection compared to facial skin, however, grades generally correlate. The median for skin was 1 and for mucosa 2 (red line). A mean difference (Oral Mucosa minus facial skin) was found to be 0.2 (95% CI -0.06059 to 0.4861). Correlation between the two tissues was robust with r = 0.72. -0.) Facial Skin versus Sentinel Skin. On average, the facial skin showed slightly higher rejection grades (mean difference sentinel skin minus facial skin -0.3). Correlation was robust with r = 0.68.

Serial 2-4 mm punch biopsies of skin and mucosa were taken when rejection was suspected or during regular follow-up outpatient visits. Tissues were formalin-fixed, paraffin-embedded, and stained with hematoxylin and eosin (H&E). All biopsies were evaluated by a multidisciplinary team at the BWH and at Yale New Haven Hospital led by senior dermatopathologists. Histological examination of facial and sentinel skin evaluated the presence and severity of rejection according to 2007 Banff working classification (5, 14). Grading of mucosal biopsies was done using criteria set for by Bergfeld et al, based on the 2007 Banff classification of skin-containing VCAs (**Figure 1**) (11). Histologically, skin (epidermis/dermis) versus mucosal (epithelium/submucosa) rejection is graded similarly. For Grade 0, there is minimal inflammation. For Grade I, there is mild perivascular lymphocytic inflammation without epithelial involvement. For Grade II, there is moderate lymphocytic inflammation and at least focal exocytosis of lymphocytes into the epidermis or mucosal epithelium. For Grade III, there is apoptosis of keratinocytes or interface dermatitis/mucositis associated with lymphocytic infiltration along the dermal-epidermal junction and focally into the epithelium. In more severe cases (Grade IV), there may be partial necrosis of the epidermis/epithelium. Normal mucosa is typically non-inflamed, without significant perivascular or interface inflammation and without apoptotic keratinocytes. In addition, clinical signs of rejection were assessed (inspection for changes from baseline such as erythema, ulcerations) and mucosa (intraoral exam to identify ulcers, enanthema and other abnormal findings). Although Banff grade I lacks specificity with respect to demonstrating effector-target cell interactions, the presence of mild perivascular inflammation is considered abnormal and in clinical context was regarded as indicating mild rejection (15).

### Statistical analysis

Data analysis was conducted using Prism 10.3.0 (GraphPad Software, San Diego, California, USA), and R-4.4.1 (R Foundation for Statistical Computing, Vienna, Austria). Diagnostic accuracy was assessed by calculating sensitivity, specificity, positive predictive value (PPV), and negative predictive value (NPV) for skin, mucosa, and sentinel biopsies. T-test was used to compare rejection grades across biopsy sites. Receiver Operating Characteristic (ROC) curves were constructed to evaluate the predictive accuracy of each biopsy site, with area under the curve (AUC) values used to quantify their discriminatory power. Rejection grades for facial skin, oral mucosa, and sentinel flap biopsies were compared using paired statistical tests and correlation analyses. A paired t-test was performed to assess whether rejection grades differed between tissue types. Pearson’s correlation coefficient (r) was used to evaluate the strength of the relationship between paired rejection grades.

## Results

### Mucosa and skin

Our study included 47 encounters at the time of combined skin and mucosa biopsy across 9 patients (for details see **Supplementary Figures 1**, **2**). The number of encounters per patient varied, reflecting individualized follow-up schedules and frequencies of clinical rejection episodes. The majority of patients had multiple encounters: Patient 3 had the highest number with 11 biopsy encounters, followed by Patient 5 with 9 encounters, Patient 7 with 8 encounters, and Patient 9 with 7 encounters. Patient 1 had 6 encounters. Two patients (Patients 6 and 2) had 2 biopsy encounters each, while Patients 4 and 8 had a single biopsy encounter each. The average postoperative month (POM) at the time of biopsy was 34.6 months, with a range spanning from 0 to 150 months. Rejection grades were assessed histologically for both skin and mucosa acquired at the same time. The average rejection grade for skin was 1.4 (range: 0 to 3), indicating mild to moderate rejection across most encounters. Mucosa, in comparison, demonstrated a slightly higher average rejection grade of 1.6. Clinical presentations of rejection varied across encounters. In many instances, histological signs of rejection were present in the absence of clinical signs or symptoms, particularly in mucosal tissues. Treatment regimens were adjusted accordingly, with interventions ranging from changes in maintenance immunosuppression to steroid boluses for more severe rejection episodes.

### Facial skin and sentinel skin

Our study included a total of 37 biopsy encounters of paired facial skin and sentinel flap across 4 patients (for details see **Supplementary Figure 3**). The number of encounters per patient varied, with patient 5 having 5 encounters, Patient 6 having 19 encounters, Patient 7 having 10 encounters, and Patient 8 having 3 encounters. The average postoperative month (POM) for the biopsies across all patients was approximately 66.5 months, with a range from 1 to 150 months. The average rejection grade for facial skin was 1.6. For sentinel skin, the average rejection grade was slightly lower at 1.3. Clinical presentation of rejection was observed in 17 encounters for facial skin, with patients showing signs such as erythema and rash. In contrast, clinical presentations were found in 10 cases of the sentinel flap.

### Comparative analysis of facial skin and mucosa

A total of 47 paired rejection grade values were analyzed for oral mucosa and facial skin (**Figure 1**). Rejection grades ranged from 0 to 3 in both tissues. The mean rejection grade for oral mucosa was 1.574 (95% CI: 1.198 to 1.951), while for facial skin, it was 1.362 (95% CI: 1.013 to 1.710). The standard deviations were 1.281 for oral mucosa and 1.187 for facial skin. The median rejection grade for oral mucosa was 2.0, while the median rejection grade for facial skin was 1. A paired t-test did not reveal a statistically significant difference between the two tissues (P = 0.1240, two-tailed, t = 1.567, df = 46). Correlation analysis was performed and we identified a strong correlation (r=0.7180, p <0.0001).

### Comparative analysis of facial skin and sentinel skin

A total of 37 paired rejection grade values were analyzed for both facial skin and sentinel skin. The rejection grades ranged from 0 to III in both tissues. The median rejection grade was 2.0 for facial skin and 1.0 for sentinel skin, with facial skin having a slightly higher mean rejection grade (1.568) compared to sentinel skin (1.270).

The mean difference (sentinel minus facial skin) was -0.2973 (see **Figure 1B**), indicating lower rejection grades in sentinel skin, this difference was not statistically significant (P = 0.0620, two-tailed). Correlation analysis revealed a strong correlation (r=0.6828, P <0.0001).

### Clinical presentation and rejection grade in facial skin, mucosa and sentinel flap

**Figure 2:**
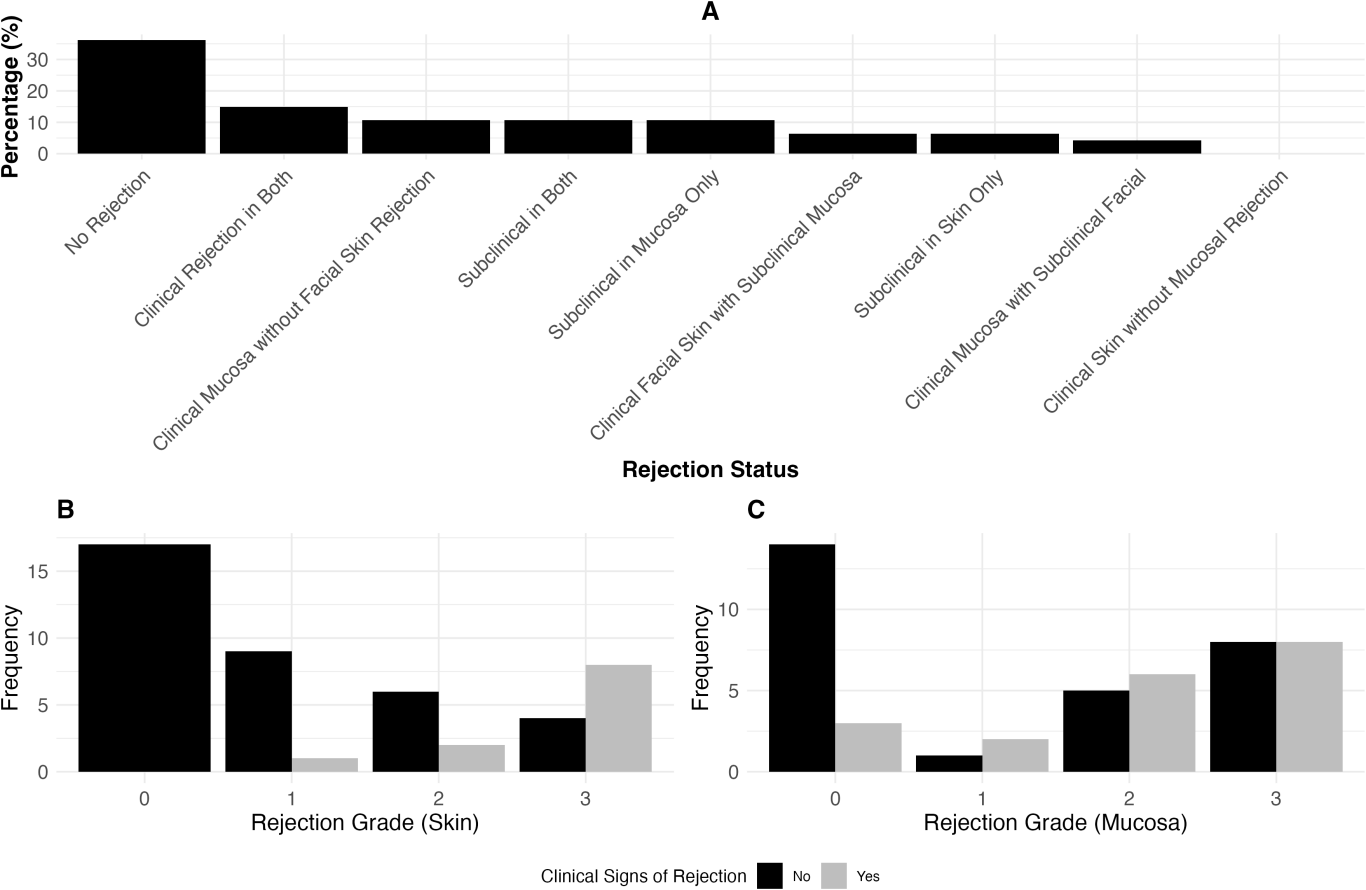
The association between rejection grade and clinical signs of rejection in skin and mucosa. **(A)** Proportion of clinical and subclinical rejection in 47 paired skin and mucosa biopsies. Clinical rejection is defined as Banff >2 with clinical signs, and subclinical rejection is Banff >2 without clinical signs. The distribution includes cases where rejection occurs in both tissues, isolated to either skin or mucosa, or no rejection at all. **(B)** Clinical presentation versus Banff rejection grades in skin biopsies (Clinical Presentation yes: Patient presented with clinical signs of rejection such as erythema). Similar to **(C)**, this panel shows the distribution of clinical signs across different rejection grades, comparing skin biopsy results. **(C)** Clinical presentation versus Banff rejection grades in mucosa biopsies. Displays the distribution of clinical signs across different rejection grades (0-3), showing the proportion of cases with and without clinical signs.

**Figure 3:**
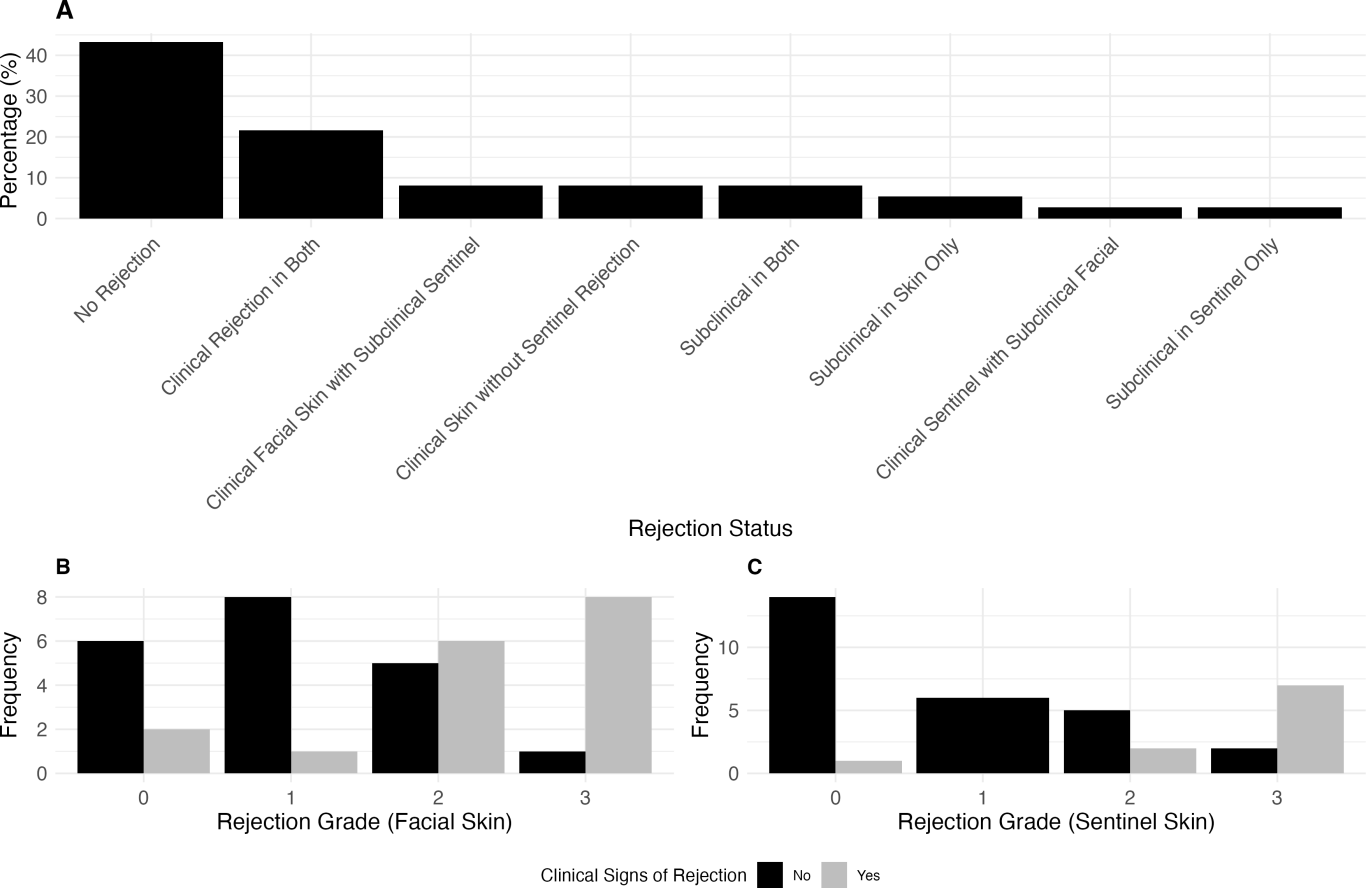
The association between rejection grade and clinical signs of rejection in facial skin and sentinel flap. **(A)** Proportion of clinical and subclinical rejection in paired facial and sentinel skin biopsies. Clinical rejection is defined as Banff >2 with clinical signs, while subclinical rejection is Banff >2 without clinical signs. The panel illustrates the distribution across cases where rejection occurs in both tissues, is isolated to either facial or sentinel skin, or where no rejection is present. In 8.1% of cases, clinical rejection was observed in the facial skin with subclinical rejection in the sentinel skin, while another 8.1% had clinical facial rejection without sentinel rejection. Clinical rejection occurred in both tissues in 21.6% of cases, while 2.7% of cases showed clinical rejection in the sentinel skin with subclinical rejection in the facial skin. No cases exhibited clinical rejection in the sentinel skin without facial involvement. 43.2% of cases had no rejection in either tissue, while subclinical rejection occurred in 8.1% of cases for both tissues, 5.4% in the facial skin only, and 2.7% in the sentinel skin only. **(B)** Clinical Presentation (yes: e.g., patient with erythema) vs. Banff Rejection Grade for sentinel skin. Rejection Grade 3 was associated with clinical signs in 7 cases, with 2 cases without clinical signs. In Grade 2, 5 cases showed no clinical signs, while 2 had clinical signs. Grade 1 had no cases with clinical signs, and in Grade 0, 1 case presented with clinical signs. **(C)** Clinical Presentation vs. Banff Rejection Grade for facial skin. Rejection Grade 3 was associated with clinical signs in 8 cases, with 1 case without clinical signs. In Grade 2, 5 cases showed no clinical signs, while 6 had clinical signs. In Grade 1, 1 case presented clinical signs, with 8 cases showing no clinical signs. In Grade 0, 2 cases showed clinical signs, while 6 had no clinical signs.

In our study of 47 paired skin and mucosa biopsies, we observed varying degrees of clinical presentation versus rejection grade in both tissues (**Figures 2B, C**). **Figure 2A** highlights the proportion of clinical and subclinical rejection across both tissues. We analyzed 37 paired sentinel and facial skin biopsies to evaluate the relationship between clinical presentation and Banff rejection grades (Results see **Figures 3B, C**). **Figure 3A**, highlights the proportion of clinical and subclinical rejection in facial and sentinel skin biopsies.

### Mucosa as a surrogate for skin rejection: diagnostic accuracy and predictive value

**Figure 4:**
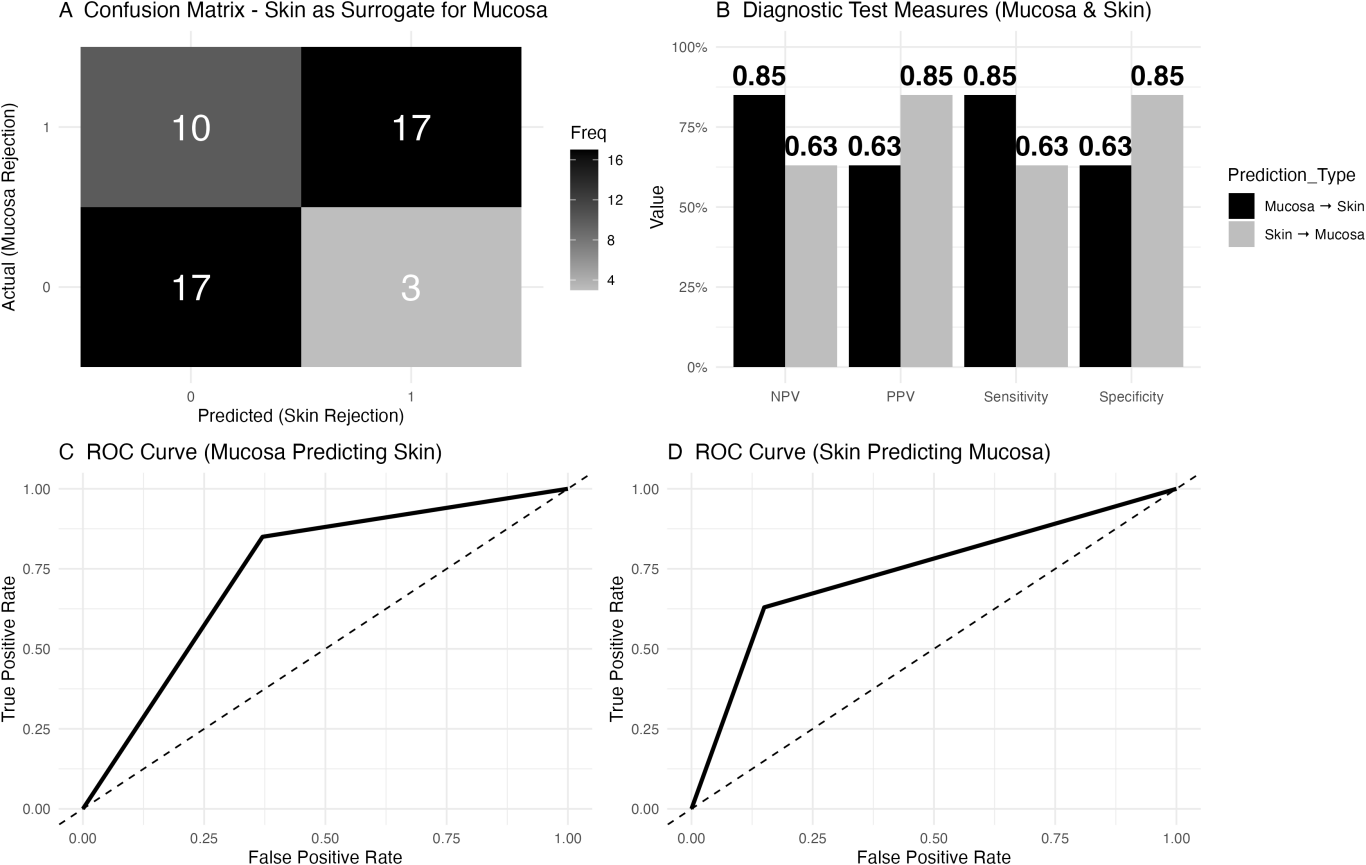
Diagnostic accuracy of mucosa to predict facial skin rejection and of facial skin to predict mucosal rejection. **(A)** Confusion matrix depicting skin as a surrogate for mucosal rejection based on histology. The matrix shows that in 10 cases, mucosal rejection was present while skin rejection was absent, while 17 cases had neither mucosal nor skin rejection. Both skin and mucosal rejection were observed in 17 cases, and in 3 cases, skin rejection was present without mucosal involvement. Rejection was defined as a Banff grade greater or equal to 2. **(B)** Diagnostic test measures for predicting mucosal rejection using skin histology. For mucosal rejection, the negative predictive value (NPV) was 0.85, the positive predictive value (PPV) was 0.63, the sensitivity was 0.85, and the specificity was 0.63. For skin as a predictor of mucosal rejection, the NPV was 0.63, the PPV was 0.85, the sensitivity was 0.63, and the specificity was 0.85. **(C)** Receiver Operating Characteristic (ROC) curve for mucosal rejection data (as a diagnostic test to predict skin rejection), with an area under the curve (AUC) of 0.74 and a 95% confidence interval (CI) ranging from 0.62 to 0.86. **(D)** Receiver Operating Characteristic (ROC) curve for facial skin to predict mucosal rejection, with an AUC of 0.74 and a 95% confidence interval (CI) of 0.62 to 0.86.

In our analysis of mucosa as a surrogate for skin rejection, the confusion matrix (**Figure 4A**) revealed that in 10 cases, mucosal rejection was present without skin rejection, while 17 cases showed neither mucosal nor skin rejection. Both mucosal and skin rejection were observed in 17 cases, and in 3 cases, skin rejection occurred without mucosal involvement. Diagnostic test measures were calculated for both mucosa and skin (**Figure 4**).

### Review of three cases with higher skin rejection grades compared to mucosa

In total, 3 encounters (6% of all paired skin and mucosal biopsies) showed skin rejection greater than mucosal rejection. These cases involved 2 different patients. One patient had 2 encounters. Case 1 (Patient 1, Postoperative Month 20): During routine follow-up, the skin biopsy showed early II rejection, characterized by mild perifollicular inflammation and lymphocyte exocytosis within the follicular epithelium. The mucosal biopsy revealed no abnormalities. The patient was given no additional treatment, continuing with scheduled belatacept infusions after transitioning from tacrolimus due to kidney injury. A repeat biopsy 2 months later showed no abnormalities.

Case 2 (Patient 1, Postoperative Month 49): During another routine visit, the skin biopsy showed rare necrotic keratinocytes within the follicular epithelium, notable Demodex mites in follicles and intraepithelial lymphocytes. No treatment was administered at that time. A repeat biopsy 2 weeks later showed no abnormalities. However, 3 weeks later, due to some erythema, a follow-up biopsy revealed spongiotic eczematous changes, suggestive of seborrheic dermatitis. The patient was prescribed ketoconazole shampoo and topical steroids.

Case 3 (Patient 2, Postoperative Month 16): This patient presented for routine follow up and a skin biopsy showed early Grade II rejection, while the concomitant mucosal biopsy showed no changes. There were no clinical signs of rejection in either tissue. Close follow-up was conducted, and a repeat biopsy 2 weeks later revealed no abnormalities.

### Review of cases with higher mucosal rejection grades compared to skin

**Figure 5:**
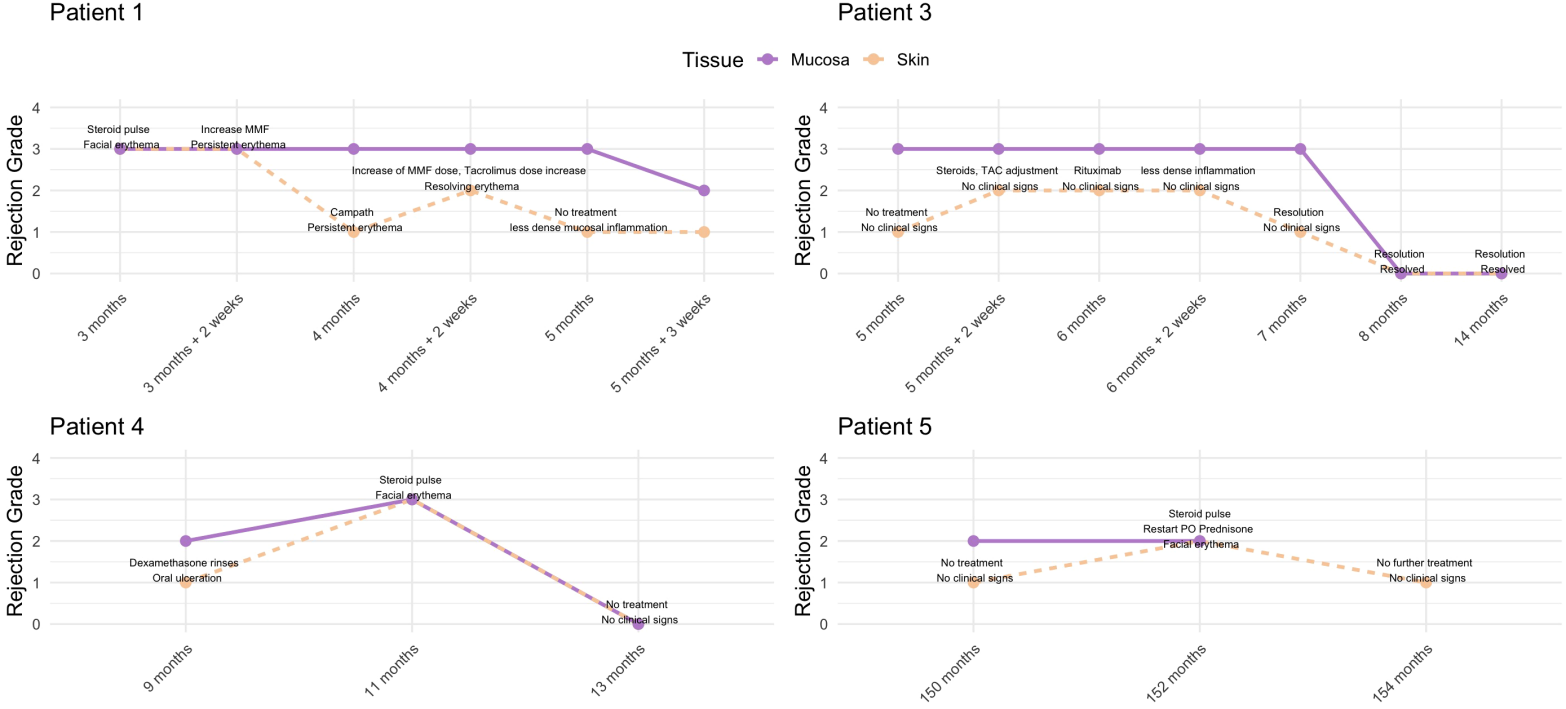
Overview of rejection episodes in patients where mucosal rejection grades were higher than skin. Patient 1 showed persistent mucosal rejection despite skin improvement, requiring alemtuzumab for resolution. Patient 3 had fluctuating rejection in skin and mucosa, with mucosal rejection persisting longer. Patient 4 developed clinical skin rejection following treatment for mucosal lesions. Patient 5 initially had no treatment but later developed skin rejection, requiring intervention.

In total, 5 encounters were noted in which mucosal rejection grades were higher than skin, involving 4 patients. Details are shown in **Figure 5**. Patient 5 initially did not receive treatment but developed skin rejection 2 months later, requiring intervention. Patient 4 received oral dexamethasone rinses for an oral lesion and developed clinical skin rejection 2 months later. Patient 3 showed grade III mucosal rejection at POM 5 without skin rejection. Two weeks later, skin rejection developed and was managed, while persistent mucosal rejection required multiple immunosuppressive adjustments, eventually leading to resolution. Patient 1 had grade III rejection in both tissues; the skin responded to steroids and adjustments to maintenance IS, but mucosal rejection persisted, necessitating further treatment with alemtuzumab. The rejection episodes resolved gradually over several weeks.

### Sentinel skin as a surrogate for facial skin rejection

**Figure 6:**
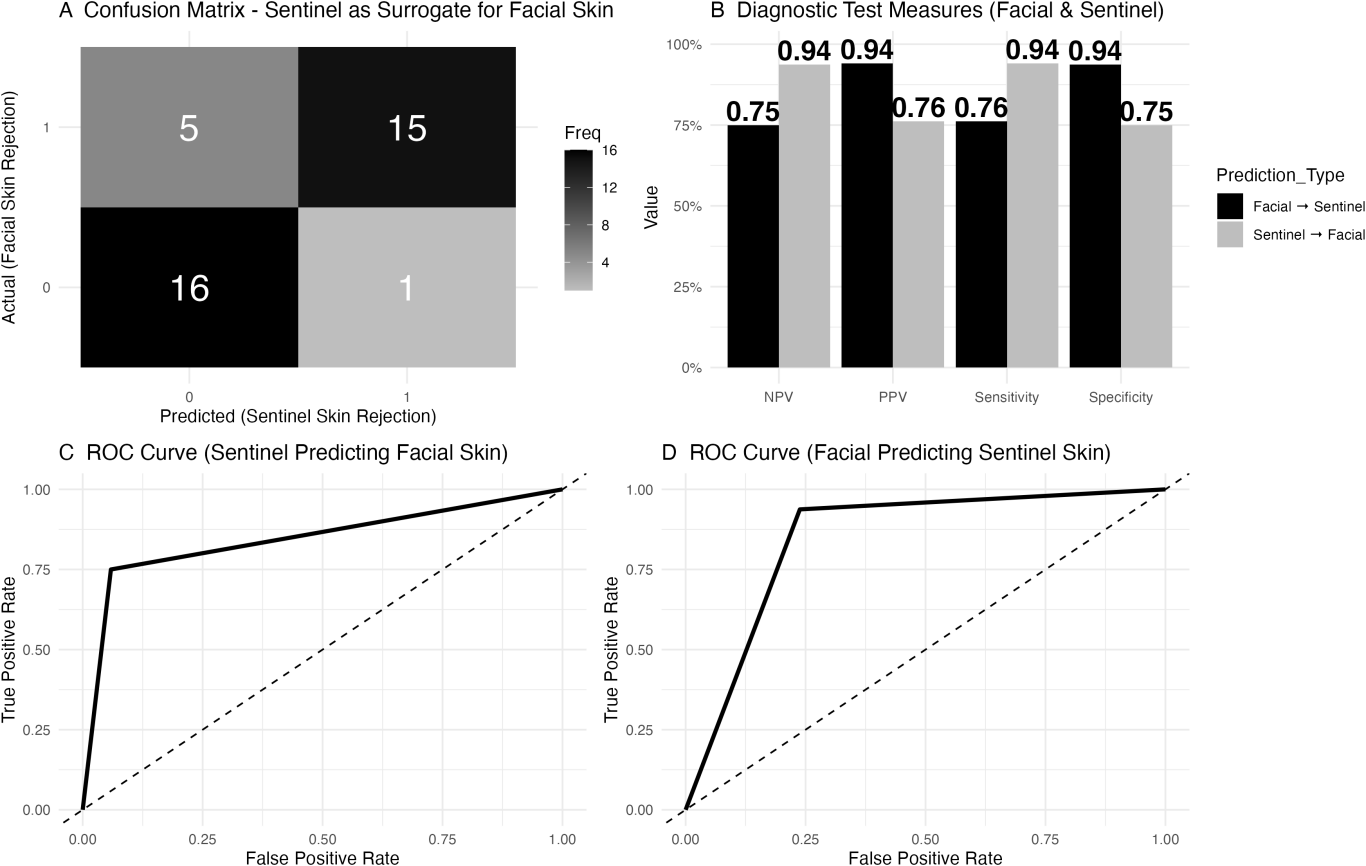
Diagnostic accuracy of sentinel skin to predict facial skin rejection. **(A)** Confusion matrix for sentinel skin as a surrogate for facial skin rejection. The matrix shows that in 5 cases, facial skin rejection was present while sentinel skin rejection was absent, while 16 cases showed no rejection in either tissue. Both facial and sentinel skin rejection were observed in 15 cases, and in 1 case, sentinel skin rejection was present without facial involvement. **(B)** Diagnostic test measures for sentinel skin as a predictor of facial skin rejection. The negative predictive value (NPV) was 0.76, with a positive predictive value (PPV) of 0.94, sensitivity of 0.75, and specificity of 0.94. **(C)** Receiver Operating Characteristic (ROC) curve for sentinel skin predicting facial skin rejection, with an area under the curve (AUC) of 0.85 and a 95% confidence interval (CI) of 0.73 to 0.96. **(D)** Receiver Operating Characteristic (ROC) curve for facial skin predicting sentinel skin rejection.

In the assessment of sentinel skin as a surrogate for predicting facial skin rejection, the confusion matrix (**Figure 6A**) revealed that in 5 cases, facial skin rejection was present without sentinel skin rejection, while 16 cases showed no rejection in either tissue. Both facial and sentinel skin rejection were observed in 15 cases, and in 1 case, sentinel skin rejection was present without facial involvement. Diagnostic test measures were calculated for sentinel skin as a surrogate for facial skin rejection (**Figure 6**).

## Discussion

Cell-mediated acute rejection in facial VCA is graded based on the degree of lymphocytic target tissue infiltration (in particular T-cells) in the context of target cell injury such as the epidermis, vasculature, and pilosebaceous units (16). This immune response is typically graded using the Banff classification. Grade III rejection is characterized by severe immune-mediated damage, including keratinocyte apoptosis, endothelial cell injury from lymphocytic vasculitis, and variably dense inflammation targeting both epidermal and vascular structures (5, 17, 18). To date, rejection of facial transplants is poorly defined and most of our mechanistic knowledge stems from skin tissue, often extrapolated from extremity transplants (8, 16, 19).

In fVCA, the chronology of tissue-specific rejection remains uncertain. Historically, skin biopsies have been used to predict rejection across all transplanted tissues. However, our data and previously published data indicate that mucosal rejection may occur earlier or more frequently, capturing rejection activity that skin biopsies alone may miss (8, 11, 19, 20). However, the lack of a dedicated classification system for rejection in mucosal tissue, supported by a robust dataset of samples and patients, remains a significant limitation in the field. Mucosa differs fundamentally from skin in its cellular composition and structural characteristics, influencing its response to rejection. For example, unlike skin, mucosa contains only mast cells with tryptase (as opposed to both tryptase- and chymase-containing mast cells) and lacks the mature barrier formed by the stratum corneum and stratum granulosum, while its submucosa demonstrates rapid, relatively scarless healing compared to the dermal sclerosis and scarring seen in skin during chronic inflammation (21, 22). Some additional mucosal-specific considerations are exemplified in other epithelium directed pathologies such as lichen planus which often demonstrates B-cell/plasma cell infiltration which has been demonstrated as a unique feature of mucosal rejection compared to skin rejection (19, 23–25). The only existing classification, developed by Bergfeld et al. in 2013, extrapolates a grading system from skin to mucosa while accounting for some tissue-specific disease manifestations (11). However, this system is based on a single patient with a short follow-up period, highlighting the need for more comprehensive studies.

In our analysis, mucosal rejection did not consistently predict subsequent skin rejection, as observed in two out of five patients, where mucosal rejection did not lead to skin rejection even after two months. In one case, mucosal rejection preceded skin rejection by two weeks, suggesting a potential temporal association. However, these findings underscore the complexity of rejection patterns, which may manifest in spatial clusters rather than uniformly across tissues and even in the same tissue.

Nonetheless, the strong correlation (r = .72) between skin and mucosa biopsy grades (graded based on the degree of lymphocytic infiltration and target cell injury) demonstrates similarities in the pathophysiology of rejection in both tissues. Clinical signs of rejection were more common in higher-grade rejection events, in particular in skin. The tissue-specific difference may be related to the more difficult assessment of potentially subtle signs of mucosal rejection on intraoral exam which is typically done without dedicated dental equipment and possibly suboptimal light source.

The correlation between skin and mucosal biopsy grades, as well as biopsy grade and clinical signs of rejection, indicates that current grading strategies are suitable for monitoring rejection activity.

Our findings suggest that mucosal biopsies are an essential element of a sensitive and comprehensive means to detect transplant-directed immune activity in facial vascularized composite allotransplantation. Notably, we identified only three instances where histologic skin rejection was diagnosed without concurrent mucosal rejection, suggesting that mucosal biopsies capture nearly all cases of skin rejection. Importantly, all 3 instances of disconcordance were not clinically relevant events; these cases support searching for any alternative explanation for cutaneous inflammation, like seborrheic dermatitis or demodicosis. Conversely, in 10 instances, mucosal rejection was present without corresponding skin rejection; furthermore, in three cases, the mucosal rejection grade was higher than that of the skin, with skin rejection matching the mucosal grade over a short time period, highlighting the importance of assessing mucosal biopsies.

This evidence positions mucosa as a sensitive tissue for detecting rejection, making it a valuable tool for rejection surveillance. This sensitivity challenges the traditional reliance on skin biopsies alone as the primary diagnostic measure, suggesting that mucosal biopsies should be integrated into routine monitoring to provide a more accurate and comprehensive assessment of transplant rejection. Furthermore, one practical advantage of mucosa over skin is that it heals quickly and with minimal scarring (26). In other types of epithelial-directed alloreactivity, mucosal biopsies were found to be more sensitive indicators of disease as well, such as intestinal biopsies over skin in assessing the severity of acute graft versus host disease (GVHD) after stem cell transplantation (27, 28). Similar to VCA rejection, epithelial cells of the skin, liver, and gastrointestinal mucosae are one of the primary targets in acute graft versus host disease. Activated CD8+ cytotoxic T-cells target stem cell niches, e.g. of the intestinal mucosae, and induce apoptosis and necroptosis (29, 30). A study performed by Narkhede et al. identified that histologic grades of mucosal (GI) biopsies had a higher correlation with the actual clinical grade of aGVHD compared to skin (28). Kohler et al. found that, although GI symptoms tend to appear later, the diagnostic accuracy of intestinal (mucosal) biopsies to diagnose aGVHD is higher compared to skin biopsy alone, which can be confounded by other non-specific dermatoses (31).

The question of whether to do both skin and mucosa must be answered. Although continuing both skin and mucosal biopsies may seem redundant, this dual approach could offer a more thorough rejection monitoring system. Based on the data, the addition of mucosal biopsies to skin biopsies is logical as it allows for the analysis of a control tissue. Mucosa may provide a clearer signal of overall rejection activity, while skin biopsies may still play a role in detecting and monitoring potentially confounding dermatologic conditions, such as seborrheic dermatitis or Demodex infestation, which can mimic rejection but do not represent true rejection events. Furthermore, performing skin biopsies will still provide insights into the development of chronic rejection and are essential in terms of understanding the biology of skin rejection which needs to be further studied (32).

Skin remains the most important tissue from an aesthetic point of view, as chronic rejection will be clearly visible if it affects the skin component. However, skin does not seem to reliably predict rejection in other tissues, as evidenced by mucosal pathology. In another study, it was also suspected that alloreactivity in fVCAs was seen in lymphatic tissue in the absence of skin pathology (7). It is also not clear at this point what clinical significance mucosal rejection has. It is perhaps inaccurate to base mucosal grading on skin criteria, and revising the grading criteria for mucosa could improve concordance between concurrent skin and mucosal biopsies. However, the mucosa may be simply a better indicator of immune activity directed at foreign donor-derived antigens. Adjusting immunosuppression based on a more sensitive tissue may lead to better outcomes and less chronic inflammation and may slow chronic rejection.

Additionally, skin biopsy results may also help identify false positive mucosal rejection values. Many potential confounders have previously been discussed by our work group, such as mechanical damage, infection, and mucosal disorders unrelated to transplant, which one needs to address prior to diagnosing mucosal rejection (8, 10). Mechanical damage should typically be evident based on a clinical exam, and infection can be suspected and is often further investigated in samples with clinically visible changes (e.g., erythema, crusting) by using specialized stains for common pathogens.

One critical consideration is that acute rejection grading system in facial transplantation (according to the 2007 Banff classification of acute rejection in skin containing VCAs) primarily relies on the presence or absence of lymphocytic infiltration, with or without evidence of end-organ injury (14, 18, 33). However, the clinical significance of acute rejection may evolve over time. It remains unclear whether early-phase acute rejection carries the same implications as acute rejection changes that we observe in later stages (e.g., after POM12) and how these acute changes can or should be distinguished from chronic rejection. Chronic rejection is traditionally characterized by findings such as fibrosis of the graft (with or without vasculopathy), and on histologic level by epidermal thinning, hyperkeratosis, follicular plugging, vascular ectasia, and sclerosis beneath the epidermal layer which are all distinct from acute rejection changes (5, 32, 34–37). The findings presented in this study provide a foundation for future research aimed at improving the definition and grading of both acute and chronic rejection. Further studies are needed to clarify whether acute rejection in the early post-transplant phase differs in clinical relevance from similar histologic changes observed later in long-term follow-up.

Another important consideration is the role of immune cell chimerism in the differential rejection patterns observed between skin and mucosa. In a recent study by our group, we observed the long-term persistence of donor-derived CD8+ T cells (16). These cells infiltrated deep dermal arteries within the graft and were found in direct apposition to chimerically populated endothelium of recipient origin. This interaction is presumed to contribute to arteriosclerotic changes in these vessels and is considered a feature of chronic rejection. The persistence of donor-derived immune cells highlights the potential relevance of immune chimerism tracking and its potential influence on mucosal and skin rejection, as well as the differences in immune activity observed in this study.

For example, in intestinal transplantation, donor-derived T cells of intestinal mucosal origin have been shown to migrate from the graft to the recipient’s bone marrow, where they selectively eliminate recipient hematopoietic cells, allowing engraftment of donor-derived passenger hematopoietic cells (38). This process is thought to establish stable macrochimerism (>4%) and promote immune tolerance toward the graft. Further investigation is needed to better understand these mechanisms in the context of facial transplantation and oral mucosal transplantation. This may help explain the differences between skin and mucosa and ultimately assist in refining rejection diagnosis guidelines.

Lastly, the utility of sentinel flap biopsies as a surrogate for facial skin rejection has been reviewed. Although sentinel biopsies were initially proposed to reduce the need for facial skin biopsies, the data show that they are not sufficiently reliable for diagnosing facial skin rejection, which may be due to their limited surface area. In five instances of clinical rejection, sentinel biopsies failed to detect signs of rejection, with a negative predictive value of 0.76. Despite a high specificity of 94%, this finding confirms that sentinel skin and facial skin likely share similar rejection mechanisms, but sentinel biopsies cannot substitute for facial skin biopsies in detecting rejection. In contrast, the negative predictive value of mucosal biopsies is higher and thus likely more suitable to accurately exclude ongoing skin rejection. Furthermore, mucosal biopsy could limit the need for facial skin biopsy, thereby limiting scar burden.

## Limitations

A key limitation is the small sample size, which is inherently constrained by the rarity of the procedure. This limited sample size restricts the generalizability of our findings, as the variability in patient responses to transplantation and rejection might not be fully captured. Additionally, the small cohort size reduces the statistical power of the study, potentially limiting the ability to detect subtle but clinically significant differences in rejection patterns across biopsy sites (skin, mucosa, and sentinel skin). Additionally, rejection grading was conducted by multiple pathologists throughout the study, and interobserver concordance was not formally assessed. However, to minimize variability, the majority of samples were reviewed and discussed in departmental conferences. Furthermore, the evaluation of mucosal biopsies was not blinded to the skin biopsy results, potentially introducing further bias. Lastly, being a single-center study, our results may reflect center-specific practices and patient populations, which could differ from those in other transplant centers. This necessitates caution when extrapolating our findings to broader populations and underscores the need for multicenter collaborations to validate and expand upon our observations.

## Conclusion

In conclusion, combining mucosal and skin biopsies provides a more sensitive and reliable method for detecting rejection in face transplants and should be considered a critical component of rejection monitoring protocols. The integration of mucosal biopsies alongside skin biopsies offers a more comprehensive approach to surveillance, ensuring that both mucosal and skin rejection are adequately captured while minimizing the risk of misinterpreting concurrent skin or mucosal conditions that are unrelated to transplantation as rejection. This dual approach could enhance early detection and improve overall transplant surveillance. Lastly, the NPV of sentinel flaps to predict facial skin rejection was poor at only 76%, missing 25% of rejection events. The transplantation of sentinel flaps solely for the purpose of immune monitoring may not be required based on the data shown here and sentinel flaps may not adequately mirror facial skin due to smaller surface size and/or different tissue composition.

## Data Availability

The original contributions presented in the study are included in the article/**Supplementary Material**. Further inquiries can be directed to the corresponding authors.

## Supplementary material

Supplementary Figure 1 Banff grades of mucosal and concordant skin samples over time (Patients 1-4).

Supplementary Figure 2 Banff grades of mucosal and concordant skin samples over time (Patients 5-9).

Supplementary Figure 3 Banff grades of facial skin and concordant sentinel skin samples over time.
