# Supplementary figures and images for: A comparative analysis of lesional skin, sentinel flap, and mucosal biopsies in assessing acute face transplant rejection
Source: Front Immunol. 2025 Apr 1;16:1562024. doi: 10.3389/fimmu.2025.1562024 (PMC11997448; doi:10.3389/fimmu.2025.1562024)

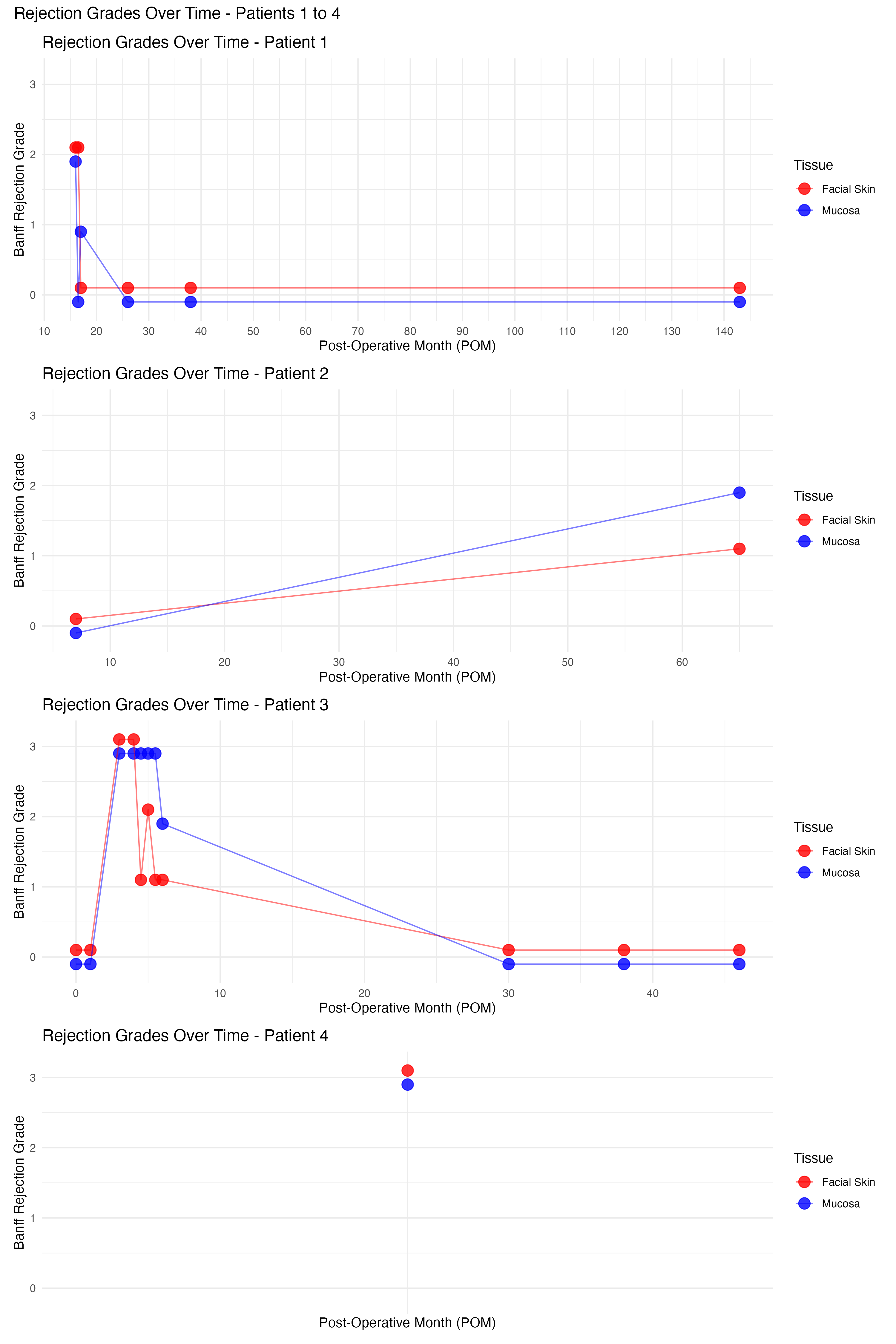

Supplement: Supplementary Figure 1 — Banff grades of mucosal and concordant skin samples over time (Patients 1-4). [file Image1.png]

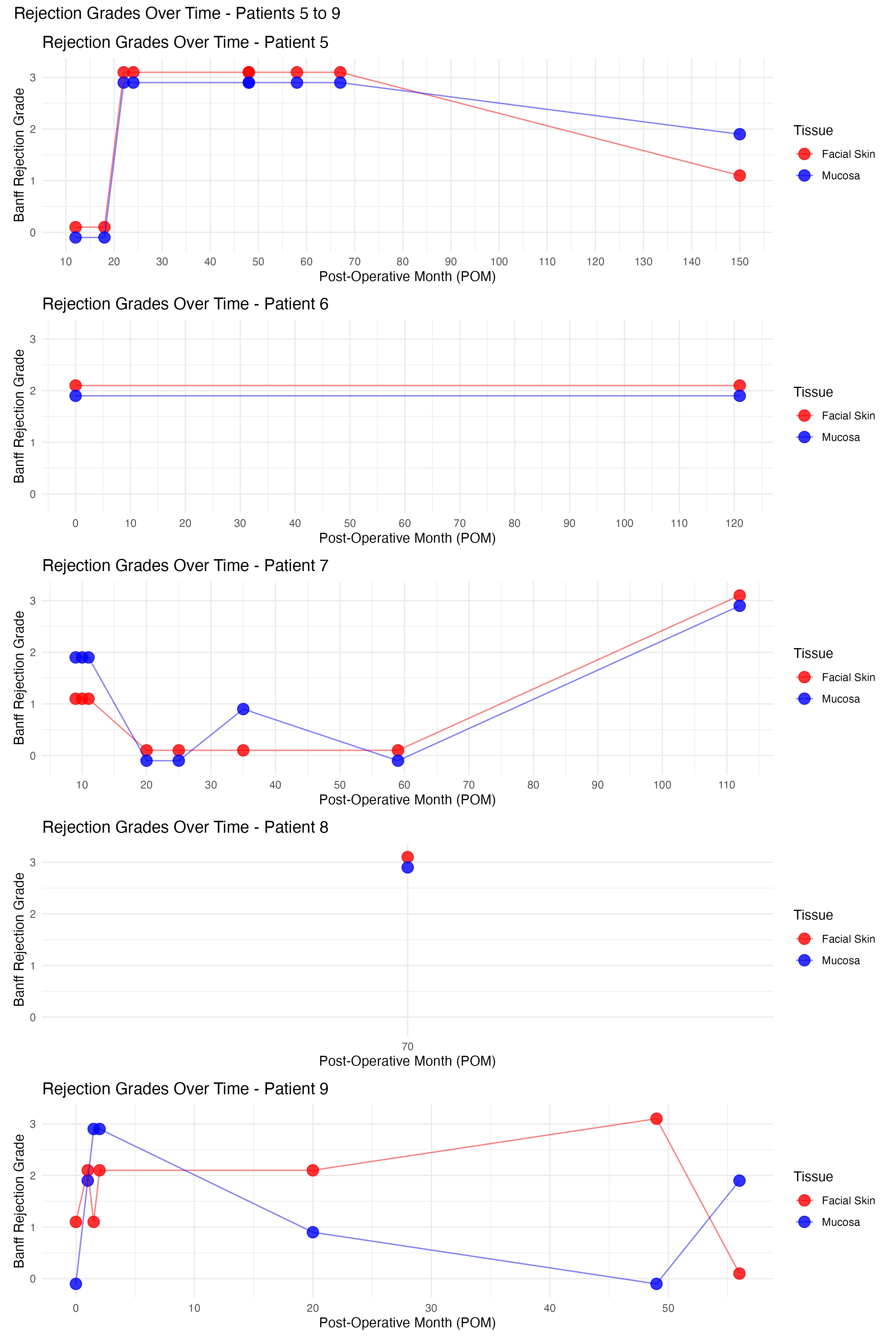

Supplement: Supplementary Figure 2 — Banff grades of mucosal and concordant skin samples over time (Patients 5-9). [file Image2.png]

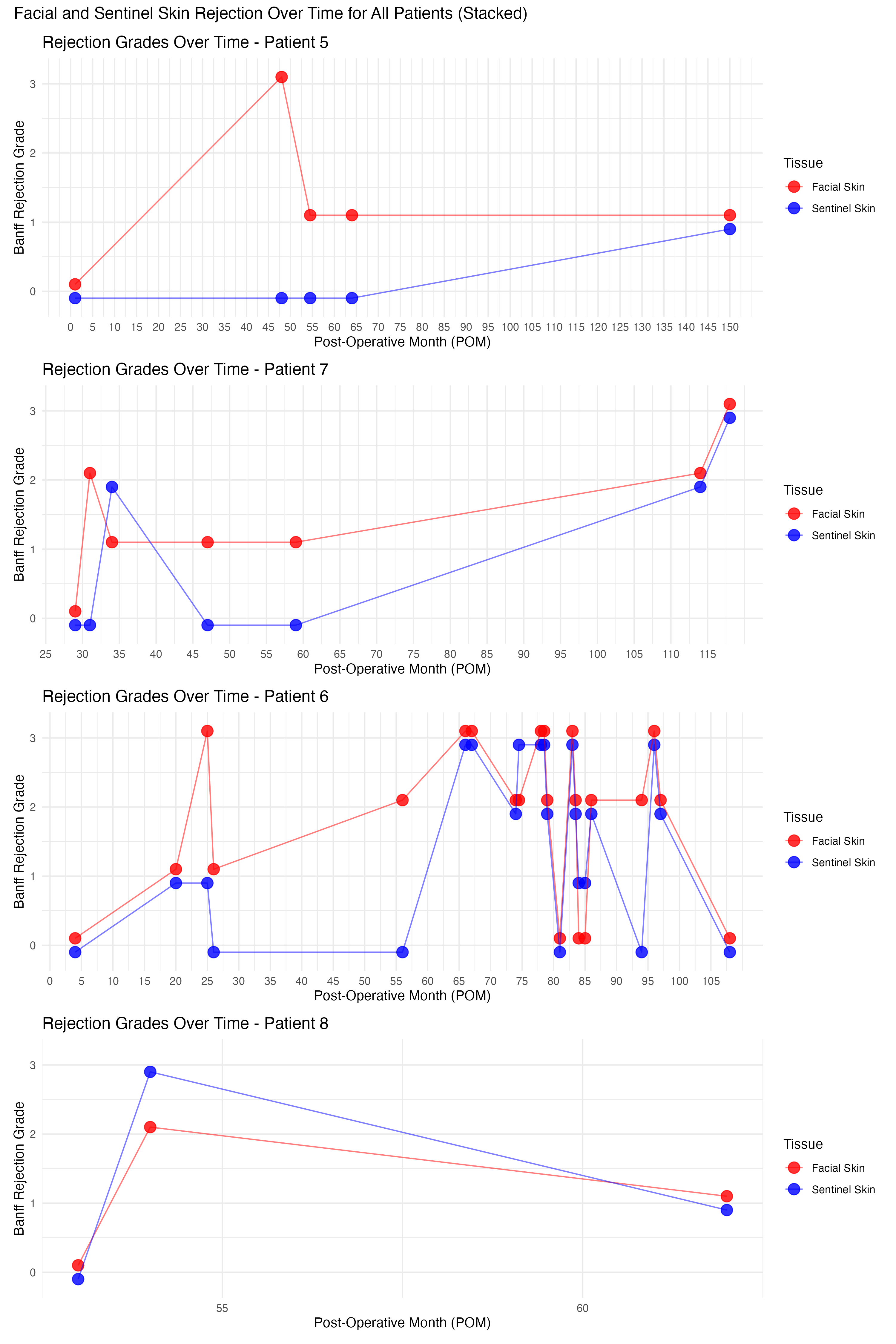

Supplement: Supplementary Figure 3 — Banff grades of facial skin and concordant sentinel skin samples over time. [file Image3.png]
